# Learning as a missing component of digital health, environment and climate change

**DOI:** 10.1038/s41746-025-02080-5

**Published:** 2025-10-29

**Authors:** Maeghan Orton, Gabrielle Samuel, Mats Blakstad, Peter Benjamin, Javier Elkin, Oscar Franco-Suarez, Felix Holl, Sarah J. Iribarren, Richard Holman Matanta, Kimberly A. Hill, Matt Hulse, Dimitrios Kalogeropoulos, Andrew Karlyn, Tanjir Rashid Soron, Anicia Santos, Temitayo Tella-Lah, Peter Drury

**Affiliations:** 1 Global Health Academy, University of Edinburgh, Edinburgh, UK; 2 Department of Global Health and Social Medicine, King’s College London, London, UK; 3 SINTEF Digital, Oslo, Norway; 4 Health Enabled, Cape Town, South Africa; 5 International Committee of the Red Cross, Geneva, Switzerland; 6 Pontificia Universidad Javeriana, Bogota, Colombia; 7 DigiHealth Institute, Neu-Ulm University of Applied Sciences, Neu-Ulm, Germany; 8 Department of Biobehavioral Nursing and Health Informatics, University of Washington, Seattle, WA USA; 9 Department of Public Health and Family Medicine, Faculty of Medicine, Universitas Hasanuddin, Makassar, Indonesia; 10 VillageReach, Seattle, WA USA; 11 World Bank, Washington, WA USA; 12 Global Health & Digital Innovation Foundation, London, UK; 13 UCL Global Business School for Health, London, UK; 14 eHealth Africa, Abuja, Nigeria; 15 Telepsychiatry Research and Innovation Network and NiHealth Ltd, Dhaka, Bangladesh; 16 Technologists for the Public Good, Sacramento, CA USA; 17 Drury Consulting, York, UK

**Keywords:** Climate sciences, Environmental social sciences, Social sciences

## Abstract

Despite its rapid advancement, digital health has little considered issues of climate change or environmental degradation. As the digital health community begin to engage with this critical issue scholars have started mapping progression in the field, typically focusing on the relationship between digital health as it applies to climate and/or environmental mitigation or climate adaptation. In this Comment, we argue that *climate and environment learning* for mitigation and adaptation constitutes a critical yet overlooked dimension intersecting mitigation and adaptation strategies, warranting deliberate attention. This learning category is the systematic and transparent approach that applies structured and replicable methods to identify, appraise, and make use of evidence from data analytics across decision-making processes related to mitigation and adaptation, including for implementation, and informs the exchange of new best practices in a post-climate era. The WHO’s Digital Health Classification framework offers a good option for ultimately formalising learning into practice. As a foundational step, however, learning needs to be conceptualised and developed into its own research agenda, organised around a shared language of metrics and evidence. We call on actors in the digital health field to develop this concrete strategy and initiate this process.

## Introduction

Despite its rapid advancement, digital health–the development and use of digital technologies and advanced data analytics to improve health, including eHealth and mHealth^1,2^–has engaged little with issues of how climate change or environmental degradation impact health. At the same time, scholars are increasingly highlighting the interrelationships between climate, environment and heath^3–6^. As the digital health community (researchers, practitioners, and policymakers) slowly begin to engage with this critical issue^7–10^, scholars have started mapping progression in the field, typically focusing on the relationship between digital health as it applies to climate/environmental degradation mitigation or adaptation^11–15^. In this short Comment–and drawing on our collective interdisciplinary expertise as digital health practitioners, Non-Governmental Organisation (NGO) representatives, policymakers, and academics who work at the intersection of digital health, environment and climate change–we argue that climate and environment learning (herein: learning) constitutes a critical yet overlooked dimension intersecting climate/environmental mitigation and adaptation strategies (herein: mitigation and adaptation), warranting deliberate attention. Learning is the systematic and transparent approach that applies structured and replicable methods to identify, appraise, and make use of evidence from data analytics across decision-making processes related to mitigation and adaptation, including for implementation, and informs the exchange of new best practices in a post-climate era. In other words, it learning entails leveraging data analysis informed by health, climate and environmental factors to generate indicators, metrics and ultimately evidence that can inform mitigation and adaptation decisions through evidence to action. Without learning, the translation of data into actionable evidence may be limited, reducing the responsiveness of evidence-informed decision-making processes essential for effective climate adaptation and mitigation.

To embed learning into health decision-making processes, we propose ultimately integrating learning metrics for mitigation and adaptation into the WHO’s Digital Health Classification. As a foundational step, learning needs to be fully conceptualised, and developed into its own research agenda organised around a shared language of metrics and evidence that can be bridged across a range of actors.

In the below sections, we describe the intersection between digital health and climate/environment adaptation and mitigation strategies. We then introduce and describe the critical yet overlooked dimension of climate and environment learning, as well as its relationship with both adaptation and mitigation efforts. We describe the WHO’s Digital Health Classification framework as a preliminary anchor to develop learning, identifying specific gaps that must be addressed to effectively incorporate learning as a fundamental component of digital health evaluation approaches. We argue that addressing this gap requires the organisation of a learning research agenda around a shared language of metrics and evidence. This approach will support the analysis and applications of evidence through Evidence to Action frameworks, which are vital to ensure that interventions are both effective and equitable in real-world settings.

## Digital health, mitigation, adaptation and the case for learning

Within current climate and environment discourse, mitigation and adaptation strategies are the predominant focus. For digital health, *adaptation focuses* on how digital health can be used to adapt healthcare system functions and community practices to the impact of climate change and reduce their vulnerability to climate-related disruptions. The aim is to prepare and support health systems for operations during climate/environment-related service delivery interruptions, increase the resilience of providers, patients and infrastructure to withstand climate and environmental events, and improve climate change and environmental resilience of the health sector through digital infrastructure^16^. Digital interventions associated with this include, for example, providing remote access to healthcare professionals, on-going clinical observations through remote patient monitoring, health worker training and patient self-care through digital means. Interventions may also include early warning systems for climate events^17^.

Alongside adaptation, digital health interventions are increasingly associated with *mitigation*. Mitigation *through* digital health interventions focuses on how digital health interventions can be used to mitigate climate change through, for example, optimising health system resource use by improving operational efficiency, and reducing the need for health service delivery practices that are implicated in environmental harms (paper/other physical material use; transport for commodities; transport for treatment)^18,19^. For example, telemedicine can mitigate environmental impacts by reducing transport-related emissions^20^. Mitigation *of* digital health interventions focuses on how digital health interventions can be designed to have minimal environmental impact: excessive technology- and data-driven approaches (rather than targeted approaches) can exacerbate digital health’s environmental impacts, and many scholars are focusing on how these impacts can be reduced through reducing energy/resource consumption and waste production associated with the use of digital technologies^21,22^, with tools being designed to help practitioners (for example, see ref. ^23^).

We introduce, and propose further refinement of, the concept of *climate and environment learning*, as a third aspect underpinning the relationship between digital health interventions, climate and the environment. *Learning*, for the purpose of this review, is the use of health, climate and environment informed data analysis to generate metrics, indicators, and standards, to develop evidence to inform mitigation and adaptation decisions through evidence to action frameworks. Learning is related to mitigation and adaptation but separate: while adaptation strategies do not always depend on climate and environment data (e.g. introducing telemedicine), they can be (e.g. tracking infectious disease outbreaks). Mitigation measures similarly are not dependent on health data (e.g. reducing transport-related emissions), though they can be informed by it (e.g. comparing environmental and health outcomes before and after a digital health policy implementation to make continuous adjustments). Learning is also related to, but distinct, from data analytics: learning draws on data analytics, though its focus is on the development of metrics and indicators to develop a shared language of evidence for mitigation and/or adaptation for an evidence to action framework. If learning is enabled in a manner which enhances evidence to action, with health, climate and environment data components clearly documented in a consistent format, then this can improve the relevance of adaptation and mitigation strategies by improving implementation design for digital health programs, and by further anchoring adaptive and/or mitigation measures in evidence. It can include, for example, informing implementation design decisions associated with best practices in deploying digital health platforms in an adaptive manner in resource limited settings or selecting appropriate digital monitoring devices for specific health conditions including heart conditions during increasingly intense heat waves^24,25^.

To achieve this, learning first requires an agreed and systematic framing, with a shared language in terms of metrics and indicators used across all data sets and analyses. This can then inform a more consistent response to both current and predicted climate and environmental impacts by bringing additional clarity to decision makers tasked with selecting, enhancing or discontinuing the use of digital health programs^26^. Previous evaluations of utilising evidence standard frameworks to inform both public health practice and policy development in response to climate have already highlighted the need for a systematic approach^27^ (such as the NICE Evidence Standards Framework^26^) suggesting the potential benefits of similar strategies for digital health programs. This is why it is important to develop learning as its own research/practitioner agenda: because it highlights the essential role of a shared understanding of evidence to allow for effective and context-sensitive implementation of digital health innovations for mitigation and adaptation.

Second, learning needs to be recognised and enabled at the policy level. A viable starting point involves expanding the WHO’s Digital Health Classification framework by adding considerations and shared measurements for climate/environment learning informed by relevant case studies. Embedding this integration of learning within an established global framework increases the likelihood of policy adoption and facilitating the necessary quickening of insight from effective mitigative and adaptive digital health interventions^28^.

## Classification frameworks as a first step for learning

As digital health interventions began to gain momentum globally as a new and novel intervention alongside of established health programs to improve health outcomes, the WHO convened a working group to formalise a taxonomy of digital and health functions in 2018 to advance the evaluation of these interventions^29^, with the potential to improve health outcomes. This classification framework, and subsequent frameworks building on it, aims to ensure clarity around the types and scale of digital health implementations, enabling policy to support data exchange and capture within this field, including capturing data around the intervention; and importantly, ensuring that captured data aligns with what is needed for continuous evaluation of implementations in terms of health outcomes across all digital health programmes. Without these details, it is difficult to continuously monitor the efficacy of a specific digital health intervention over time, and in a similar manner between countries and health programs^30^.

Climate/environment learning requires a similar interdisciplinary partnership framework including climate and environmental experts alongside of digital health and health practitioners to understand key indicators, data sources and measures in place to evaluate digital health programs focused on digital climate adaptation and mitigation interventions. By enhancing an established classification framework such as the WHO one, this can allow for a move from siloed approaches where the necessary expertise to evaluate and understand the intersection of climate and digital health is not present in the same framework, thus delaying enhancements in learning. This makes it difficult to develop a shared language for best practice. However, at present, there are a number of challenges to achieving this.

## Climate and environment learning: current challenges and future directions

Incorporating learning as a conceptual dimension into the digital health-climate-environment nexus introduces additional complexities to both designing and evaluating digital health interventions. A fundamental limitation is the widespread dispersion and limited accessibility of existing environmental data, especially in Low- and Middle-income Country contexts, which can lead to prioritisation of High Income Country learning, as well as present bias in datasets. Another limitation is the persistent fragmentation of patient data and the limited scaled adoption of agreed health standards to allow for interoperability and integration of environmental data into digital health^31^, such as the Fast Healthcare Interoperability Resource that allows exchange of patient-level data. Data fragmentation can slow the pace of learning and the confidence in evidence generated. Finally, there is a need to illustrate an investment case for incorporating climate and environmental data into routine and ongoing electronic health architecture planning processes to bring cohesion to digital health implementations across different care services^30^.

Furthermore, there is current ambiguity about what data and metrics are even needed to measure known climate and environmentally induced health impacts; how to develop metrics to account for the rapidly dynamic changes in climate and weather patterns; and how to develop a rigorous approach to identify new and unknown health risks due to climate change^32^. While an increasing body of scholarship and analysis is starting to explore these issues, this body of work lacks a unifying framing, meaning that there is no shared language in terms of health, climate and environmental measures, aligning data sharing and impact modelling practices within this approach, and no unifying agreements and alignment on indicators to quantify and attribute the impact of specific interventions, nor scale digital health interventions of known efficacy.

**Fig. 1:**
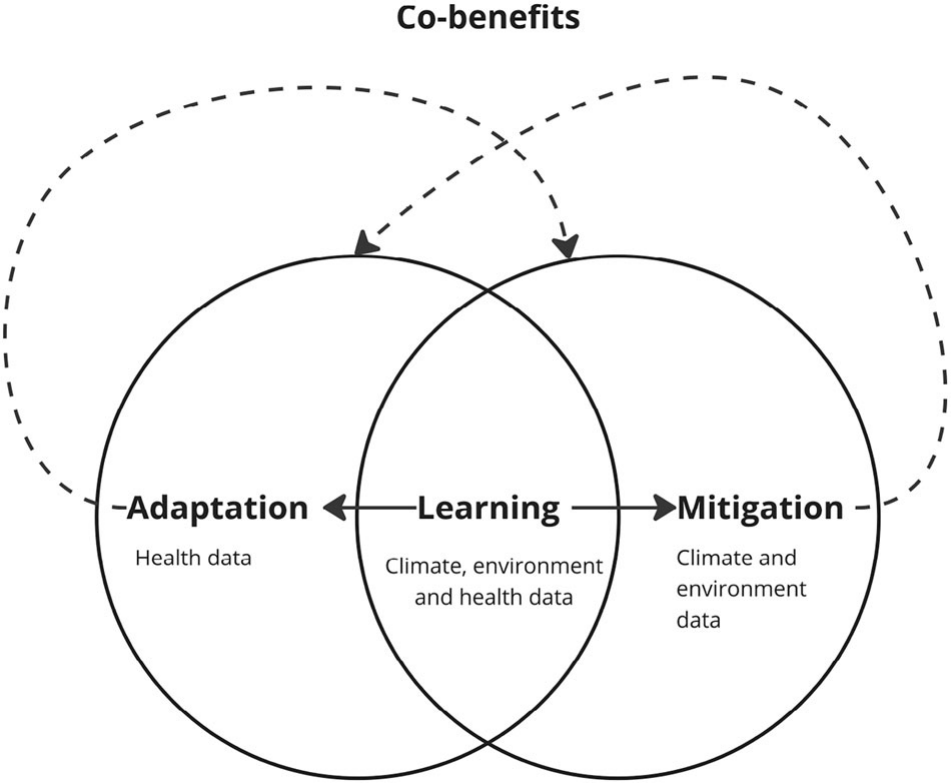
Schematic to represent how climate/environment learning intersects climate adaptation and climate mitigation. Climate/environment learning intersects both climate/environment adaptation and mitigation. Climate/environment adaptation and mitigation can have co-benefits, for example, telemedicine can improve healthcare adaptation and decrease transport emissions associated with healthcare appointments.

Finally, unintended consequences of focusing on the importance of integrating climate/environment learning into digital health systems are often largely ignored, such as the possible over-reliance on climate/environment data, which could bias digital systems in ways that overlook socio-economic drivers associated with poor health outcomes (See Fig. 1).

It is vital that these challenges are addressed to enable a quickened pace of learning in line with the pace of change due to climatological and environmental changes and resulting implications on human health. Determining how to systematically collect, standardise and share digital/climate/environment data as climate learnings, and how to relate this to health outcomes, and to develop a shared language to translate these insights into actionable practice will require collaboration across community, industry and health programs through a research agenda^32,33^.

As a starting point, we need to spend more time conceptualising what is included in the learning aspect of digital health, climate and environment, and defining the research and practitioner agenda. To begin this, available environmental and climate data sets, in terms of duration of measures, and location and granularity of data sets, need mapping. This will help advance a more consistent best practice approach around what is being measured, using what metrics, and how this relates to health outcomes. To achieve this will require the fostering of a cross-sectoral and interdisciplinary approach to sharing of knowledge and data within this new research field of digital health, climate and environmental learning.

## Data Availability

Data sharing is not applicable to this article as no datasets were generated or analysed during the current study.
